# Effectiveness of psychological intervention based on implicit theory combined with virtual reality in patients with unruptured intracranial aneurysm receiving their first cerebral angiography after flow-diverter stent implantation

**DOI:** 10.3389/fpsyt.2026.1697452

**Published:** 2026-02-16

**Authors:** Yu-juan Wang, Min Gao, Ke-xin Jiang, Wang Feng, Xiao-lin Wang

**Affiliations:** Second Affiliated Hospital, Chongqing Medical University, Chongqing,, China

**Keywords:** cerebral angiography, flow-diverterstent implantation, implicit theory, psychological nursing, unruptured intracranial aneurysm, virtual reality

## Abstract

**Background:**

To investigate the efficacy of psychological intervention using implicit theory combined with virtual reality (VR) in patients with unruptured intracranial aneurysm (UIA) undergoing their first cerebral angiography (CA) after implantation of a flow-diverter stent.

**Methods:**

One hundred and four patients with UIA who underwent their first CA six months after flow-diverter stent implantation in our hospital between October 2022 and October 2024 were enrolled by convenience sampling. The participants were then randomly divided into a control group (n=52) and an experimental group (n=52). The control group received routine nursing intervention, while the experimental group was given both routine nursing and psychological intervention based on implicit theory combined with VR. The levels of anxiety, physiological stress indicators, and treatment compliance were compared between the two groups.

**Results:**

Repeated-measures ANOVA revealed significant group × time interactions for anxiety (SAS: F(2, 204) = 267.89, *P* < 0.001, ηp² = 0.724), treatment confidence (VAS-TC: F(2, 204) = 101.36, *P* < 0.001, ηp² = 0.498), and implicit cognition (IAT: F(2, 204) = 598.74, *P* < 0.001, ηp² = 0.854). Post-intervention (T1) and pre-procedure (T2), the experimental group showed significantly lower SAS scores and higher VAS-TC and IAT scores than the control group (all *P* < 0.001). Notably, the experimental group’s mean IAT score at T2 (80.1 ± 4.5) exceeded the pre-specified threshold for positive implicit cognition (65 points). During the angiography, the experimental group also demonstrated superior physiological regulation (higher baroreflex sensitivity and heart rate variability indices, all *P* < 0.001), better examination tolerance (fewer body movements, lower pain scores, *P* < 0.001), and higher procedural compliance (accuracy of instruction execution: 92.3% vs. 67.5%, *P* = 0.001) compared to the control group.

**Conclusion:**

Implicit theory combined with VR can significantly reduce intraprocedural stress responses and increase cooperation during diagnostic and therapeutic procedures, enhancing long-term compliance through the reconstruction of cognitive structures, optimizing autonomic nerve regulation, and improving pain tolerance. The findings provide a multi-dimensional evidence-based reference for the use of psychological intervention in UIA patients following flow-diverter stent implantation.

## 1 Introduction

Unruptured intracranial aneurysm (UIA) is a potential cause of cerebrovascular accidents and has a global prevalence of approximately 3.2% (1, 2). In China, the incidence rate of UIA among individuals aged 35 and over is 7.0%, with a 1.2–8.2% risk of rupture of UIAs with diameters > 5 mm each year (3, 4). Aneurysm rupture can cause subarachnoid hemorrhage (SAH), with an associated mortality rate of over 50%, while survivors may experience severe neurological dysfunction (5). Beyond focal neurological deficits, survivors of aneurysmal SAH frequently experience persistent neuropsychiatric complications, most commonly anxiety, depression, cognitive dysfunction, headaches, seizures, and sexual dysfunction, which substantially affect quality of life and merit routine screening and follow-up support (6). Flow-diverter stent implantation has become the preferred treatment for medium-to-large-sized aneurysms, as the stent can guide blood flow and exert a positive influence on endothelialization of the aneurysm neck, resulting in a five-year complete occlusion rate of up to 82.3% (7–9). Flow-diverter stents treat aneurysms by altering neck hemodynamics. They divert inflow away from the sac, creating intrasaccular stasis and progressive thrombosis, while the device’s low-porosity scaffold supports endothelialization across the aneurysm neck to reconstruct the parent artery.

Nevertheless, cerebral angiography (CA), the gold standard of efficacy evaluation, faces significant challenges, including patient anxiety and frequent body movements during the procedure (10–12). While these issues are currently addressed by training and monitoring vital signs, patient anxiety surrounding stent effectiveness and technology remains a problem (13). Although the use of cognitive behavioral therapy (CBT) and relaxation training can reduce procedural anxiety, they often do not meaningfully shift patients’ implicit beliefs (14–16). At the same time, standard 2D angiography provides limited, patient-friendly visualization of stent endothelialization, leaving cognitive reconstruction incomplete. Beyond CBT and relaxation strategies, short-acting pharmacologic anxiolysis is often used around angiography. Benzodiazepines (e.g., midazolam) reduce anticipatory anxiety and produce anterograde amnesia but carry risks of respiratory depression and delayed recovery; while flumazenil can reverse effects, reversal is not a substitute for safe, titrated dosing (17). Opioids are frequently paired for analgesia yet add risks of hypoventilation and nausea, especially in combination with benzodiazepines. Propofol offers rapid onset/offset and excellent tolerance but is dose-dependently associated with cardiorespiratory depression and, at deeper levels, loss of verbal responsiveness that can impair cooperation and real-time neurological assessment (18). Dexmedetomidine can provide “cooperative” sedation with minimal respiratory depression, though bradycardia and hypotension are not uncommon and onset may be slower (19). Because sedation exists on a continuum, inadvertent deepening increases adverse events and reduces responsiveness, underscoring the appeal of non-pharmacologic approaches that can lessen sedative requirements while maintaining stillness and cooperation.

Virtual reality (VR) has emerged as a feasible non-pharmacological tool to mitigate anxiety and improve patient experience in medical settings. Prior clinical studies in perioperative and preprocedural contexts suggest that immersive VR, delivered as calming distraction, guided relaxation, or structured procedural preparation, can reduce state anxiety and distress while improving satisfaction and perceived control (20, 21). Importantly, these effects are clinically relevant for invasive or image-guided procedures that require sustained stillness and timely execution of instructions, where anxiety-related movement and poor cooperation may compromise procedural efficiency and image quality (22, 23). Mechanistically, VR may attenuate anxiety through attentional capture and “presence” (competing with threat-related cognitions), affect regulation via multisensory relaxation, and anticipatory exposure/education that improves predictability and controllability of the procedure (24). However, existing VR interventions in medicine are often not explicitly theory-driven, and evidence remains limited regarding whether VR programs tailored to a specific interventional context and patient concern can simultaneously improve emotional outcomes, behavioral cooperation, and physiological stress responses during angiography-like procedures.

Proposed by Dweck in the 1990s, implicit theory describes a fundamental belief in the reconstruction of human attributes (e.g., IQ, personality). This influences personal perceptions of the world and their corresponding responses and emphasizes the implicit view (also known as the mindset) of individuals (25). In the angiography context, a growth-oriented implicit theory may help patients reframe uncertainty and discomfort as manageable and improvable, thereby supporting coping, cooperation, and adherence. Therefore, we developed an implicit-theory–guided VR intervention tailored to patients undergoing CA at 6-month follow-up after flow-diverter treatment for UIA. We hypothesized that this theory-driven, procedure-tailored VR program would reduce anxiety and pain, improve cooperation-related behaviors during CA/DSA, and attenuate physiological stress responses.

## 2 Subjects and methods

### 2.1 Subjects

A total of 104 patients with unruptured intracranial aneurysms (UIA) were enrolled in this study. All patients underwent flow-diverter stent implantation at a Grade 3A hospital between October 2022 and October 2024 and received their first cerebral angiography six months postoperatively. The study protocol was implemented and data collection was conducted after approval by the hospital’s Ethics Committee (Approval No. 2022-194). Written informed consent was obtained from all patients prior to enrollment.

Inclusion criteria: ① Diagnosed with UIA and having undergone flow-diverter stent implantation; ② Undergoing re-examination using CA for the first time six months after surgery; ③ Participating voluntarily in the study; ④ Conscious and able to cooperate with the subsequent interventions and investigations. Exclusion criteria: ① Do not meet the requirements for CA re-examination; ② The presence of cognitive impairment or inability to communicate and cooperate with subsequent interventions and investigations.

Sample size calculation: G*Power 3.1 was used to estimate the sample size based on the inter-group differences in Self-Rating Anxiety Scale (SAS) scores. The significance level was set at α=0.05, with a statistical power 1-β of 0.8 and an effect size d of 0.6. The calculation showed that 48 cases were required in each group, and the sample size was expanded to 52 cases/group to allow for a 10% dropout rate, resulting in a total sample size of 104 cases. The participants were randomly divided into the control group (n=52, routine nursing intervention) and the experimental group (n=52, routine nursing intervention + intervention with implicit theory combined with VR). The general characteristics (e.g., sex, age, severity) of the two groups did not differ significantly (*p* > 0.05, **Table 1**).

**Table 1:** General information on the control and experimental groups.

Variables	Control group (*n* = 52)	Experimental group (*n* = 52)	t/χ²	*P2*
Age (years, x ± s)	55.3 ± 8.2	56.1 ± 7.9	t= -0.51	*p* = 0.61
Sex (cases, %)				
Male	14 (26.9%)	17 (32.6%)	χ²=0.04	*p* = 0.85
Female	38 (73.1%)	35 (67.4%)		
Education (cases, %)				
Elementary school and below	15 (28.8%)	16 (30.8%)	χ²=0.06	*p* = 0.91
Junior high school or vocational school	20 (38.5%)	19 (36.5%)		
High school and above	17 (32.7%)	17 (32.7%)		

### 2.2 Randomization and blinding

Eligible participants who provided written informed consent were randomly assigned in a 1:1 ratio to the control or intervention group. The random allocation sequence was generated using IBM SPSS Statistics 28.0 (IBM Corp., Armonk, NY, USA) by an independent statistician who was not involved in participant recruitment, intervention delivery, or outcome assessment. Allocation concealment was ensured using sequentially numbered, opaque, sealed envelopes containing the group assignments. After enrollment, a research nurse opened the next envelope in sequence to determine the assignment and implemented the allocated intervention according to the protocol. The investigators responsible for screening and enrolling participants had no access to the randomization sequence.

Due to the nature of the VR-based intervention, blinding of participants and the clinical staff delivering the intervention was not feasible. To minimize detection bias, outcome assessors and data collectors were blinded to group allocation throughout data collection and analysis. Participants were instructed not to disclose their group assignment to the assessors.

### 2.3 Intervention

#### 2.3.1 Control group

The control group received routine nursing intervention, comprising: (1) Basic education: A paper brochure combined with verbal explanation was used to introduce the purpose, procedure, method, position requirements, and potential risks of CA examination to reduce the patient’s examination anxiety. The duration of the explanation was approximately 15 minutes; (2) Daily care: The vital signs of the patients were monitored, with administration of medications as prescribed by the doctor and close observation of any adverse effects of the medications; (3) Review instructions: Patients were informed of the importance of regular review (e.g., brain CT, MRA, CA) after flow-diverter stent implantation to monitor aneurysm embolization and the risk of recurrence.

#### 2.3.2 Experimental group

**Figure 1:**
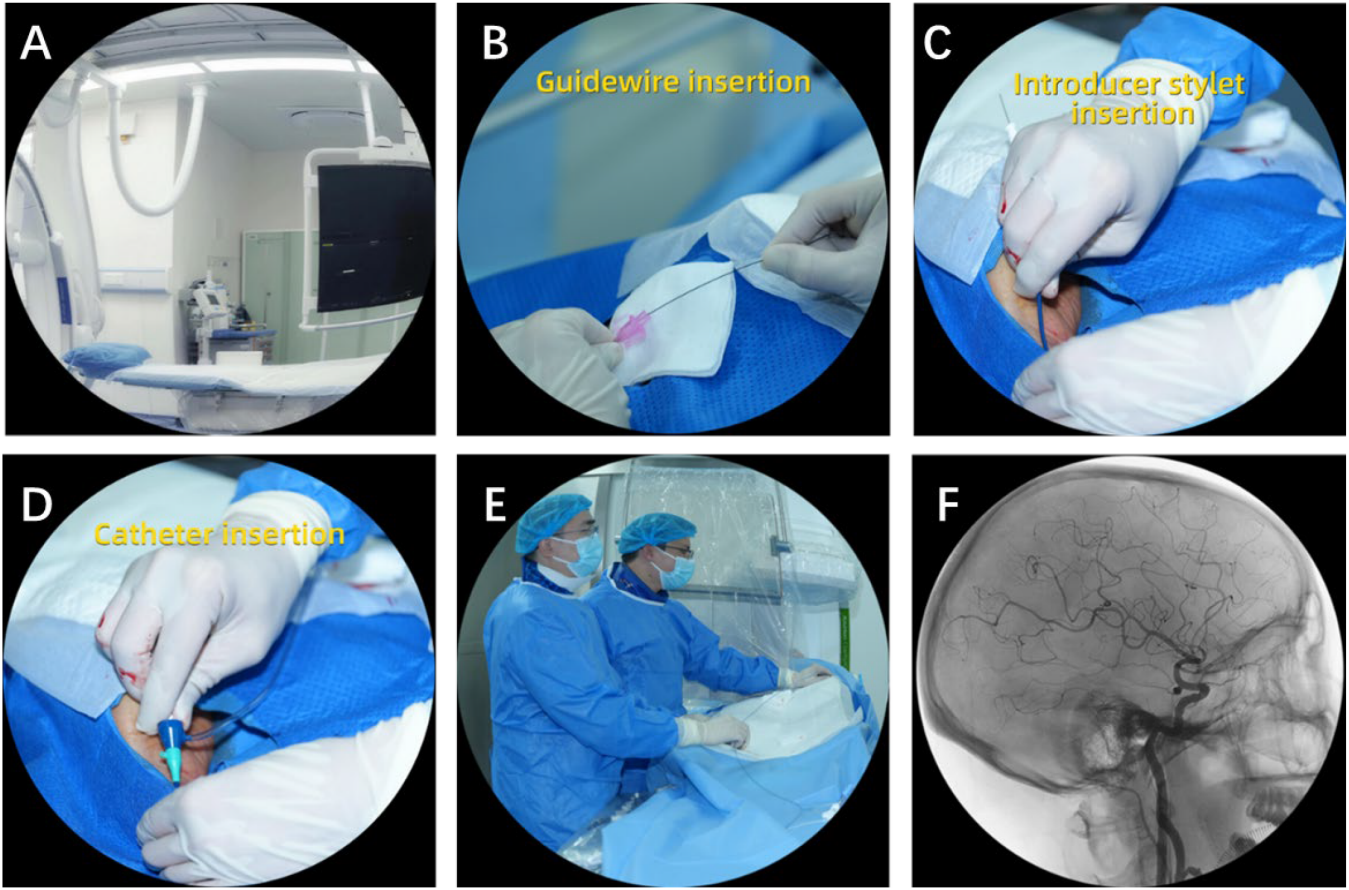
Schematic illustration of the VR educational narrative guidance module. **(A)** Angiography suite setup, providing an overview of the procedural environment; **(B)** guidewire insertion; **(C)** introducer stylet insertion; **(D)** catheter insertion; **(E)** operative team performing the endovascular procedure using standard techniques; **(F)** representative digital subtraction angiography (DSA) image used to explain vascular anatomy and device deployment principles.

The experimental group received routine nursing care plus an implicit-theory–guided VR psychological intervention one day before procedure (**Figure 1**). This interval was chosen to balance recency (next-day recall and anxiety reduction) with brief consolidation time and operational feasibility within standard workflows. This involved:

1. Establishment of an intervention team: The team comprised one associate chief physician from the Department of Neurosurgery, one attending physician, one head nurse from the interventional operating room, two nurses from the Department of Intervention, and one VR trainer. The associate chief physician reviewed the program plans and provided medical guidance and supervision. The attending physician reviewed the medical accuracy of the VR content, guided assessments of treatment compliance, and optimized the intervention protocols. The head nurse coordinated the work of the team to ensure that the implicit theory-based VR education process was aligned with the interventional specialty care process, oversaw training of the nursing staff, and managed the patient feedback information. Nurses from the Department of Intervention were responsible for instructing patients in VR operations, patient education, and the collection of feedback, while the VR trainer was responsible for the development and technical maintenance of the VR.

2. Design of VR modules and functions: Based on the “cognitive scaffold” principle of construction in implicit theory, the patients’ implicit cognition of the disease and treatment was reshaped using multimodal visual, auditory, and tactile inputs. The implicit theory-based VR content was determined after in-depth discussion and analysis by the VR team and addressed the psychological and compliance-related problems experienced by patients after flow-diverter stent implantation. The specific modules included:

- Educational narrative guidance: This module combined standardized educational video segments with computer-generated 3D visualizations to provide patients with an intuitive overview of the procedure (**Figure 1**). A patient-specific 3D intracranial vascular model was generated from the participant’s imaging data to support intuitive understanding of aneurysm location, parent vessel anatomy, and the structural relationship between the flow-diverter and the vessel. The module used guided narration and visualizations to explain the principle of flow diversion and the expected course of healing in patient-friendly language.
- Metaphorical neural recoding training: Based on a fusion of the physical model and VR, 3D printing was used to customize the anatomical model of the brain using the MRI data of the patient, with the UIA aneurysm represented as a removable red marking area while the stent was denoted as a protective mesh cover. The patient could practice interactive placement of the protective mesh (stent) over the red area (aneurysm) while observing how the stent altered the direction of the blood flow. The tactile-visual-auditory linkage exercise reinforced the patient’s awareness of the protective effect of the stent and based on the concept of mirror neurons, guided the patient to describe the principles of the operation after the procedure.
- VR simulation of the CA examination process: A vascular endoscopy simulation system based on Unreal Engine 5 and Unity 2021.3 LTS: UE5 was used for high-fidelity medical visualization (vessel reconstruction, dynamic blood-flow rendering), whereas Unity was used for the interactive procedural-simulation and haptic-feedback modules due to its strengths in real-time interaction and device integration. The patient entered the virtual vascular environment by controlling the “nanorobot” through the joystick, enabling the detection of the endothelial coverage area of the stent in real-time. When the coverage rate reached a threshold (e.g., ≥ 70%), the system provided the feedback of “stent in good condition” and triggered an encouraging animation. Nociceptive-behavioral correlation training was also conducted. A visualization of the approaching puncture needle was inserted at a critical point in the examination (e.g., femoral artery puncture), with a simulation of mild pain by application of a wristband haptic feedback device (with adjustable amplitude). The patient was guided to adjust their breathing to reduce their fearful anticipation of the real examination.
- Strengthening and consolidating the system: Individualized analysis of the patient’s behavioral performance during the VR training (e.g., operation time, number of errors, task completion patterns) was conducted to identify issues such as “over-concern about the risk of stent migration” or “underestimation of the rate of endothelialization”, and the attending physician addressed these specific misconceptions on the day of surgery. Meanwhile, a scenario-based quiz (interactive multiple-choice questions) was used to reinforce learning motivation and identify potential issues.

3. Nurse training: Before the implementation of the intervention, two training sessions were organized by the VR trainer and the head nurse to ensure that the nurses from the Department of Intervention were proficient in the VR application. The training content covered the basics of implicit theory, construction and operation of the VR equipment, interpretation of the DICOM image data, and the use of 3D printing. The nurses were required to familiarize themselves with wearing and adjusting the VR equipment and to understand the operational procedures of the VR equipment and software, as well as to familiarize themselves with the relevant skills through hands-on experience. The training focused on the key process of metaphorical neural recoding training. After completion of the training, the nurses were able to operate the VR equipment independently, guide patients in understanding the 3D model of intracranial blood vessels and the principles and processes involved in flow-diverter stent implantation, as well as mastering the application of implicit theory.

4. Intervention: Using the combination of implicit theory and VR for psychological intervention, the nurses from the Department of Intervention provided the patients with detailed instructions on the correct wearing of the VR headset and associated precautions, informing them of possible adverse effects (e.g., motion sickness, eye fatigue), and emphasizing the need to communicate any experience of discomfort. During the intervention, the attending physician monitored the patient’s vital signs, listened to the patient’s complaints, and ensured that the patient was comfortable. At the end of the intervention, the nurses provided additional explanations and demonstrations based on the patient’s responses and the imaging process to enhance the patient’s understanding and improve CA compliance.

The intervention lasted approximately 40–45 minutes. The structured session comprised: (1) introduction and headset fitting/safety briefing (5 min); (2) implicit-theory–guided psychoeducation (5 min); (3) educational narrative guidance with vascular model visualization (10 min); (4) metaphorical neuro-recoding training (10 min); (5) VR simulation of the CA/DSA workflow (10 min); and (6) consolidation with scenario-based questions and targeted clarification (5 min).

#### 2.3.3 VR intervention

The VR intervention was delivered using an Oculus Quest 2 head-mounted display (Meta Platforms, Inc.), a standalone wireless headset (refresh rate 90 Hz) with integrated Oculus Insight inside-out six-degrees-of-freedom (6-DoF) motion tracking. Participants interacted with the environment using handheld controllers. The headset and controllers were disinfected before and after each use with 75% medical alcohol wipes, and each participant used a single-use sterile sweat-absorbent facial cover to minimize cross-contamination and improve comfort.

The VR content was custom developed for this study through a collaboration between the research team, the hospital’s medical engineering department, and a computer science laboratory. Patient-specific vascular geometry was reconstructed from CTA DICOM data using Mimics Medical 23.0 (Materialise NV). The intervention used pre-scripted simplified animations consistent with general flow-diversion principles to visually demonstrate the core concept that inflow is redirected away from the aneurysm sac toward the parent vessel following flow-diverter deployment. The VR program included interactive modules. Participants manipulated virtual objects via controllers, and the system provided immediate visual and auditory feedback based on interaction completion and accuracy. This allowed individualized pacing while maintaining a standardized content structure across participants. During and immediately after the intervention, a nurse monitored and recorded VR-related adverse symptoms including nausea, dizziness and visual fatigue. In the experimental group, 2 participants (3.8%) reported mild dizziness that n after a brief rest; no participants withdrew due to VR discomfort.

#### 2.3.4 Sedation and analgesia protocol

All patients underwent cerebral angiography under a standardized light-sedation and analgesia protocol. To minimize operator-related bias, medications were administered by interventional physicians who were blinded to group allocation. In accordance with institutional guidelines, each patient received intravenous midazolam at a dose of 0.02–0.03 mg/kg prior to the start of the procedure to achieve conscious sedation. Additional fentanyl (0.5–1.0 μg/kg) was administered as needed to alleviate puncture-related discomfort. Review of anesthesia records showed no significant differences between the control group and the intervention group in the total doses of midazolam (control: 1.3 ± 0.3 mg vs. intervention: 1.2 ± 0.3 mg, p = 0.127) or fentanyl (control: 35.2 ± 8.1 μg vs. intervention: 33.8 ± 7.6 μg, p = 0.361). This indicates that the intra-procedural pharmacological sedation and analgesia exposure was comparable across groups.

### 2.4 Indicators

#### 2.4.1 Evaluation of psychological indices

The following psychological assessment scales were administered to the patients before the intervention (time T0), immediately after the intervention (time T1), and 1 h before the CA examination (time T2) using a combination of Wenjuanxing and paper questionnaires to assess anxiety levels, disease perception, confidence in treatment, and changes in implicit cognition:

1. SAS: The SAS contained 20 items, covering somatization symptoms (e.g., palpitations, dizziness) and psychological anxiety (e.g., tension, fear). A 4-point Likert scale (1=none or occasional, 4=persistent) was used, and the total score was multiplied by 1.25 for conversion to a standardized score ranging from 25 to 100 points. The Cronbach’s α of the SAS was 0.83-0.86, indicating good reliability and validity.
2. Visual Analog Scale for Treatment Confidence (VAS-TC): The VAS-TC had no sub-items and was scored using a 10 cm horizontal scale (0 = no confidence, 10 = complete confidence). This was converted to a score between 0 and 10 points after the patient had marked their level of confidence in the treatment on the scale.
3. Implicit Association Test (IAT): The IAT consisted of two sets of associations contrasting disease threat (e.g., “aneurysm risk”) with treatment efficacy (e.g., “stent safety”). Standardized scores were calculated using the improved D-algorithm, based on differences in response latencies between congruent and incongruent categorization tasks (26). Raw D values (ranging approximately from –2 to +2) were then linearly transformed to a 0–100 scale using the standard rescaling formula (D + 2)/4×100, with higher scores indicating stronger implicit identification with treatment safety. In accordance with commonly used effect-size conventions for D values in the IAT literature, D values in the vicinity of 0.65 are typically interpreted as reflecting a strong implicit association; on the transformed 0–100 scale, this corresponds to approximately 66 points. Therefore, a score of ≥ 65 points was adopted as an operational criterion for a strong positive implicit cognition pattern in this study. The Cronbach’s α for the IAT scale was 0.78.

#### 2.4.2 Monitoring of physiological indices

The heart rate variability (HRV) of the patient and the number of shifts in position due to discomfort during the imaging procedure were recorded by a cardiac monitor. At the end of the examination, the pain level at the puncture site was assessed using a Numerical Rating Scale (NRS). The VR system did not incorporate real-time physiological sensing and no wearable physiological interface or motion-capture module was used during the VR session. To assess the intervention’s effect on intra-procedural stress, HRV and BRS were recorded only during the CA/DSA procedure using the routine clinical monitoring setup and were not recorded during the VR exposure. Prior to HRV analysis, raw ECG signals were subjected to visual inspection and automated correction to identify and remove ectopic beats and movement-related artefacts. HRV indices were computed from 5-minute stationary segments selected during the CA/DSA procedure. The selected segments were band-pass filtered (0.03–0.40 Hz), and standard time-domain and frequency-domain algorithms were used to derive HRV metrics. BRS was calculated from synchronized beat-to-beat cardiovascular signals using the same artefact-screened segments.

#### 2.4.3 Evaluation of behavior and compliance

The duration of breath-holding during surgery, the precision of position maintenance, and the number of cases in which the patient refused to continue due to emotional breakdown were recorded to evaluate the patients’ compliance. Long-term patient compliance was assessed by the percentage of patients who took the initiative to make a follow-up appointment within three months and the percentage who failed to complete the scheduled six-month follow-up.

### 2.5 Handling of missing data

Analyses were conducted under an intention-to-treat (ITT) framework, with all 104 randomized participants retained in their originally assigned groups. No missing data occurred during the VR intervention, the CA procedure, or the psychological assessments at T0, T1, and T2, allowing the repeated-measures ANOVA for psychological outcomes to be performed on a complete dataset. Missing data were present only for the 6-month follow-up compliance outcome (control group: 16 cases; intervention group: 4 cases). These follow-up data were analyzed on an available-case basis within the ITT framework, and sensitivity analyses indicated that the observed group differences remained robust despite attrition.

### 2.6 Statistical analysis

Data were analyzed with SPSS 28.0. Continuous variables were assessed for normality using the Shapiro–Wilk test. Normally distributed data are presented as mean ± standard deviation (SD), whereas non-normally distributed data are presented as median (interquartile range, IQR). Categorical variables are presented as counts (percentages). Baseline characteristics were compared using independent-samples t-tests for normally distributed continuous variables, Mann–Whitney U tests for non-normally distributed continuous variables, and χ² tests for categorical variables. Psychological outcomes measured repeatedly at T0, T1, and T2 were analyzed using repeated-measures ANOVA (RM-ANOVA), with time as the within-subject factor and group as the between-subject factor, focusing on the group × time interaction. When the interaction effect was significant, simple-effects analyses were conducted to examine between-group differences at each time point and/or within-group changes across time. For between-group comparisons using t-tests, Cohen’s d and its 95% CI were calculated. For categorical outcomes, risk differences (RD) or odds ratios (OR) with 95% CIs were reported as appropriate. For repeated-measures ANOVA models, partial eta-squared (ηp²) was reported as the effect size for main and interaction effects. Given that RM-ANOVA framework for the psychological outcomes (SAS, VAS-TC, IAT) provides omnibus control of type-I error for the pre-specified time, group, and interaction effects, no further correction was applied; for the multiple independent physiological and behavioral comparisons collected during CA (HRV parameters, BRS, movement counts, and pain scores), FDR correction (q < 0.05) was used, and the reported p-values represent the adjusted results. P < 0.05 was considered statistically significant.

## 3 Results

### 3.1 Psychological indices

#### 3.1.1 SAS scores

The RM-ANOVA showed significant main effects of time (F(2, 204) = 185.32, P < 0.001, ηp² = 0.645) and group (F(1, 102) = 253.47, P < 0.001, ηp² = 0.713), as well as a significant group × time interaction (F(2, 204) = 267.89, P < 0.001, ηp² = 0.724) (**Table 2**). Simple-effects analyses indicated no between-group difference at baseline (T0; P = 0.653). However, the intervention group had significantly lower SAS scores than the control group at T1 and T2 (both P < 0.001). Over time, the intervention group showed a sustained reduction from T0 to T2 (all within-group comparisons P < 0.001), whereas the control group showed no significant change across the three time points (P = 0.071). The significant group × time interaction indicates that the intervention produced a superior trajectory of anxiety reduction over time compared to routine care.

**Table 2:** Repeated-measures ANOVA results for SAS and VAS-TC across T0, T1 and T2 (mean ± SD).

Outcome	Group	N	T0	T1	T2	Time effect (F, p, ηp²)	Group effect (F, p, ηp²)	Group × time (F, p, ηp²)
SAS	Control	52	57.8 ± 5.9	55.1 ± 6.3	57.1 ± 5.7	F=185.32 p<0.001 ηp² =0.645	F=253.47 p<0.001 ηp² =0.713	F=267.89 p<0.001 ηp² =0.724
	Intervention	52	58.3 ± 6.2	36.5 ± 5.1	21.3 ± 7.2			
VAS	Control	52	7.2 ± 4.7	8.3 ± 5.2	7.9 ± 3.6	F=92.15 p<0.001 ηp² =0.474	F=87.42 p<0.001 ηp² =0.462	F=101.36 p<0.001 ηp² =0.498
	Intervention	52	6.9 ± 4.5	13.5 ± 6.4	21.1 ± 7.2			

SAS, self-rating anxiety scale; VAS, visual analog scale.

#### 3.1.2 VAS-TC scores

For treatment confidence, RM-ANOVA demonstrated significant main effects of time (F(2, 204) = 92.15, P < 0.001, ηp² = 0.474) and group (F(1, 102) = 87.42, P < 0.001, ηp² = 0.462), and a significant group × time interaction (F(2, 204) = 101.36, P < 0.001, ηp² = 0.498) (**Table 2**). There was no baseline difference between groups at T0 (P = 0.720). At T1 and T2, the intervention group reported significantly higher VAS-TC scores than the control group (both P < 0.001). Within-group analyses showed a stepwise increase in VAS-TC scores in the intervention group across time (all P < 0.001), while scores remained stable in the control group (P = 0.215). Thus, the significant interaction reflects that the intervention not only boosted confidence at specific time points but also fostered a progressive, time-dependent increase in treatment confidence, a pattern absent in the control group.

#### 3.1.3 IAT scores

For implicit cognition, RM-ANOVA revealed significant main effects of time (F(2, 204) = 512.34, P < 0.001, ηp² = 0.834) and group (F(1, 102) = 945.21, P < 0.001, ηp² = 0.903), as well as a highly significant group × time interaction (F(2, 204) = 598.74, P < 0.001, ηp² = 0.854) (**Table 3**). Simple-effects analyses indicated no between-group difference at baseline (T0; P = 0.229). The intervention group showed significantly higher IAT scores than the control group at T1 and T2 (both P < 0.001). The intervention group exhibited a sustained increase across time (all P < 0.001), and at T2 the mean IAT score (80.1 ± 4.5) exceeded the prespecified positive-cognition threshold (65 points), whereas no significant change was observed within the control group (P = 0.102). The highly significant group × time interaction, with a large effect size, confirms that the intervention specifically and powerfully reshaped patients’ implicit attitudes toward treatment safety, an effect not observed under standard nursing.

**Table 3:** Repeated-measures ANOVA results for IAT across T0, T1 and T2 (mean ± SD).

Group	*n* (cases)	T0	T1	T2	Time effect (F, p)	Group effect (F, p)	Group × time (F, p)
Control	52	40.2 ± 4.2	42.5 ± 6.1	44.2 ± 4.9	F=512.34 p<0.001 ηp² =0.834	F=945.21 p<0.001 ηp² =0.903	F=598.74 p<0.001 ηp² =0.854
Intervention	52	41.2 ± 4.7	62.1 ± 7.2	80.1 ± 4.5			

IAT, implicit association test.

### 3.2 Physiological indices

#### 3.2.1 Heart rate variability

During the CA examination, the time domain index (SDNN), high-frequency power (HF), and vagal tone index (HFnu) in the experimental group were significantly higher than those in the control group. In addition, the LF/HF ratio in the experimental group was significantly lower than that in the control group (*p* < 0.001, **Table 4**).

**Table 4:** Heart rate variability parameters in the experimental and control groups (`x±s).

Variables	Control group	Experimental group	*t-*value	*p-*value
*N* (cases)	52	52		
SDNN (ms)	52.4±10.7	65.3±12.1	5.92	<0.001
HF (ms²/Hz)	36.7±8.1	48.2±9.3	7.01	<0.001
HFnu	42.1±7.5	58.3±6.9	11.8	<0.001
LF/HF	1.8±0.6	1.2±0.4	6.33	<0.001

SDNN, time domain index; HF, high-frequency power; HFnu, vagal tone index; LF, low-frequency power.

#### 3.2.2 Baroreflex sensitivity

During the CA examination, the BRS in the experimental group was significantly higher than that in the control group (*p* < 0.001, **Table 5**).

**Table 5:** Between-group comparisons of BRS and protection-threshold attainment rate.

Group	N (cases)	BRS (ms/mmHg)	Protection threshold compliance rate (≥10 ms)
Control	52	8.7 (7.2, 10.1)	15.4%
Intervention	52	12.3 (10.8, 14.0)	82.7%
*t-*value		Z = -8.92	/
*p-*value		<0.001	Fisher test, *p* < 0.001

BRS, baroreflex sensitivity.

#### 3.2.3 Examination tolerance (body movements and pain)

The results showed that the number of body movements during the CA examination was significantly higher in the control group compared to the experimental group, while the pain scores in the experimental group were significantly lower than those in the control group (both *p* < 0.001, **Table 6**).

**Table 6:** Between-group comparisons of examination tolerance outcomes.

Group	N (cases)	Number of body movements (n)	Pain score (score)
Control	52	3.0 (3.0, 4.0)	5.0 (4.0, 6.0)
Intervention	52	1.0 (0.0, 1.0)	2.0 (1.0, 3.0)
*t-*value		Z = -9.12	Z = -8.45
*p-*value		<0.001	<0.001

### 3.3 Behaviors and compliance

#### 3.3.1 Treatment compliance

During the CA examination, the accuracy of executing instructions was significantly greater in the experimental group relative to the controls (*p* < 0.001), while the rate of examination interruption due to pain, physical discomfort, or psychological factors was 12.5% in the control group, compared to 0% in the experimental group, representing a statistically significant difference (*p* < 0.001, **Table 7**).

**Table 7:** Treatment compliance in the control and experimental groups.

Group	N (cases)	Accuracy of instruction execution	Examination interruption rate
Control group	52	67.5%	12.5%
Experimental group	52	92.3%	0%
χ²		11.9	/
*p-*value		0.001	Fisher test, *p* = 0.006

#### 3.3.2 Long-term compliance

At six months after the CA examination, it was evident that intervention using implicit theory combined with VR was effective in enhancing patient treatment initiative, and the experimental group had a significantly lower dropout rate at six months after CA examination compared with the control group (*p* < 0.001, **Table 8**).

**Table 8:** Long-term compliance in the control and experimental groups.

Group	N (cases)	Six-month proactive review appointment rate	Six-month dropout rate
Control group	52	54.2%	31.2%
Experimental group	52	86.7%	8.3%
χ²		14.7	8.9
*p-*value		<0.001	0.003

## 4 Discussion

### 4.1 Cognition reconstruction in patients with UIA undergoing first CA examination after flow-diverter stent implantation using psychological intervention based on implicit theory combined with VR

Prior work grounded in implicit/mindset theory has linked growth-oriented beliefs with better cognitive–affective regulation and has suggested potential involvement of prefrontal–limbic circuitry and threat reactivity; immersive, multimodal interventions may further support emotion regulation by reducing the salience of threat-related cues (27, 28). In the present study, the IAT scores in the experimental group were significantly higher than those in the controls, while the level of anxiety in the experimental group was also significantly reduced relative to the controls, suggesting a shift in implicit attitudes accompanied by lower reported anxiety during the peri-procedural period (*p* < 0.001, **Tables 2**, **3**). Additionally, VR simulation could activate the mirror neuron system in patients, enabling transfer of the procedural memories established in the virtual environment to the real medical scenario, together with strengthening of the dorsolateral prefrontal cortex to regulate the pain (29–31). In the present study, patients in the experimental group used a VR module to simulate catheter-based endovascular navigation. Using a joystick, they guided a “nano-robot” through the planned steps. The module was designed to enhance procedural understanding and reduce anxiety, thereby decreasing fear-related movement during imaging and improving treatment compliance. Additionally, by reinforcing the ability of the patients to regulate pain, both the pain perception and pain scores during puncture were significantly reduced, thus effectively improving the patients’ confidence during treatment (*p* < 0.001, **Table 6**).

### 4.2 Effects of implicit theory combined with VR on autonomic nerve regulation and intraprocedural stress responses

The neural-visceral integration model suggests that increased BRS can further enhance the buffering capacity of the central nervous system against cardiovascular stress through the solitary nucleus-parabrachial nucleus-paraventricular nucleus pathway (32, 33). In the present study, the HRV time-domain indices and frequency-domain parameters in the experimental group were increased in correspondence with the psychological intervention of implicit theory combined with VR, suggesting increased vagal tone and inhibition of sympathetic activity, forming a virtuous circle (*p* < 0.001, **Table 4**). Additionally, the BRS values in the experimental group were significantly higher than those in the control group. This may be attributed to enhanced buffering capacity and tolerance to treatment stress induced by the solitary nucleus-parabrachial nucleus-paraventricular nucleus pathway, resulting in reduced frequency of intraprocedural body movements and fewer examination interruptions (*p* < 0.001, **Tables 5**, **6**).

### 4.3 Driving role of psychological intervention based on implicit theory combined with VR in the long-term behavior of patients after flow-diverter stent implantation

Self-efficacy theory states that the accumulation of successful experiences represents a core driver of behavioral change (34). In the present study, the CA examination process was simulated by VR, and an encouraging animation was triggered when the endothelial coverage reached the standard, representing an immediate feedback mechanism enabling a repeated experience of the protective effects of the stent in VR and the accumulation of successful experiences. This type of positive reinforcement may activate the dopamine reward circuit in the ventral striatum, further reinforcing implicit cognition and influencing the behavior of the patient. The accuracy of instruction execution in the experimental group was 92.3%, with an 86.7% rate of proactive appointment booking for review after six months, both of which were significantly higher than those in the control group (both *p* < 0.001, **Tables 7** and **8**). Overall, implicit theory combined with VR-based psychological intervention was associated with better cooperation-related behavior during CA and improved follow-up adherence indicators in our study.

### 4.4 Clinical translation to alternative aneurysm treatments

Anxiety, anticipatory pain, and movement during catheter angiography are not unique to FD recipients; therefore, the core elements of our intervention, implicit-belief reframing, graded exposure to the examination sequence, paced-breathing with real-time interoceptive focus, and nociceptive-behavioral linkage, are likely transferable across treatment strategies. What varies is the content of the VR psychoeducation: for stent-assisted or simple coiling, visualizations would substitute coil mass compaction/parent-artery patency for endothelialization; for microsurgical clipping, modules would emphasize clip position and vessel/perforator preservation; for patients followed primarily with MRA/CTA, the simulated environment would reproduce MRI/CT scanner cues and instruction sets rather than catheter navigation. We anticipate similar directionality of effects (reduced anxiety and movement, improved cooperation), but magnitudes may differ because procedure length, device-specific concerns, and local sedation practices vary across modalities. A multi-arm trial comparing tailored VR modules across FD, coiling, and clipping pathways is warranted to quantify generalizability.

While the observed pattern of reduced anxiety/pain, improved cooperation, and altered autonomic indices is consistent with proposed cognitive–affective regulation pathways, the specific neurobiological mechanisms cannot be directly established in this study because no neuroimaging or neurophysiological measures were collected. Accordingly, these mechanistic accounts should be interpreted as plausible hypotheses that warrant direct testing in future work.

### 4.5 Limitation

In addition to the constraints already noted, our cohort comprised only adults with unruptured intracranial aneurysms treated with flow-diverter devices and undergoing first 6-month DSA, which limits generalizability to patients managed with alternative strategies (coiling, stent-assisted coiling, microsurgical clipping) and to follow-up pathways that rely on MRA/CTA. We also excluded patients with a history of aneurysmal SAH; extending trauma-informed tailoring of the intervention to post- aneurysmal SAH populations warrants dedicated evaluation. Another limitation is that the control condition was not matched to the VR intervention in duration or level of engagement, so non-specific factors such as increased attention or the novelty of VR may have contributed to the observed effects. Moreover, participant and intervention-provider blinding was not feasible due to the nature of the VR intervention, which may have influenced subjective outcomes despite assessor blinding. Furthermore, we did not conduct a standardized usability assessment, and future work should quantify usability and engagement alongside clinical endpoints. The psychological module was delivered ~24 hours before angiography for pragmatic and theory-informed reasons, but we did not compare alternative delivery intervals (same-day, ≥72-hour, or booster schedules). These factors should be addressed in future multi-arm, modality-specific studies to better define external validity and optimal timing. Finally, although all patients received a standardized light-sedation and analgesia regimen administered under blinded conditions, and no significant differences were observed between groups in midazolam or fentanyl dosing, individual differences in pharmacological sensitivity may still have influenced physiological, pain, or movement-related outcomes. Therefore, residual confounding from sedative or analgesic effects cannot be fully excluded.

## 5 Conclusion

A triad model of “cognitive reconstruction-neuromodulation-behavioral output” was constructed based on implicit theory combined with VR, providing a novel theoretical framework for psychological intervention in patients with UIA undergoing their first CA examination after flow-diverter stent implantation. Compared with CBT, the proposed model realized psychological-physiological-behavioral multidimensional intervention by targeting implicit belief systems with multisensory integration training, which has significant value in clinical translation. However, due to the single-center nature of this investigation, there was a lack of neuroimaging data for direct demonstration of neurological changes in patients who received psychological intervention. Future studies should combine multimodal functional neuroimaging techniques to enable the dynamic tracking of changes in prefrontal-limbic functional connectivity during psychological intervention. Additionally, personalized VR content could be developed to provide patients with individualized assistance by dynamic adjustments of the intervention system.

## Data Availability

The original contributions presented in the study are included in the article/supplementary material. Further inquiries can be directed to the corresponding author.
